# Notes and News

**Published:** 1900-12-29

**Authors:** 


### Notes and News.

The annual subscription dance in connection with the Middlesex Hospital will be given at the Empress Rooms, Kensington, on February 11th.

At the urgent request of the military authorities, the Imperial Yeomanry Hospitals Committee have decided to prolong the duration of all their hospitals until April. The whole of the medical staffs and nursing sisters have consented to renew their agreements, and further orderlies are being despatched. Dr. Washbourn remains at Pretoria, and Majors Stonham and Hale are with the Field Hospital and Bearer Company.

The Report of Dr. Alfred Lingard, the Imperial Bacteriologist for India, shows that in spite of the destruction of the laboratory at Muktesar much useful work has been done. The principal work of the year was that undertaken in the hope of controlling an extensive epidemic of rinderpest in the North-Western Provinces. Up to April 1900 over 47,000 c.cm. of serum had been distributed for preventive inoculation. Attempts were also made to deal with the plagues of locusts which do such damage to the crops in India, and for this purpose tubes of a fungus which had been found pathogenic to locusts in South Africa were distributed throughout nearly all parts of India. Much work was also done in connection with an outbreak of glanders-farcy.

Many people who would gladly help forward the movement in favour of the use of leadless glaze for potteryware are in this difficulty, that, however much they may ask for it, they cannot "see that they get it." We wish we could help them to distinguish the right sort from the wrong, but unfortunately it seems impossible. It appears, on the one hand, that the fact of ware being stamped "leadless glazed" is by no means to be taken as a guarantee that the glaze is really devoid of lead; and, on the other, that much cheap pottery which is really "leadless" does not bear that motto in consequence of "its being ao cheap that it would not pay to stamp it." The chemist, of course, eats off the glaze with hydrofluoric acid and tests the product for lead, but the ordinary non-chemist will, we are afraid, for long to come have to take the dealer's word for it.

The Medical Faculty of the University of London has elected Dr. Lauriston E. Shaw, who is Assistant Physician and Dean of the Medical School at Guy's Hospital, Secretary of the Faculty.

There is at present a considerable prevalence of smallpox in New York, about 60 cases having been reported. The *New York Medical News* says that at least 100,000 people have been vaccinated, and that sore arms are the rule throughout the city.

In giving evidence in the case of Cunningham *v.* The *Daily Express*, Limited, Professor Sidney Martin is reported to have said that "it was the practice of medical men when they made a discovery in medicine to announce it at once to the world. If anyone did not wish his discovery to be known at once, he would place a record of it in a sealed envelope and forward it to the President of the Royal Society." What has the poor College of Physicians done, we wonder, that its President should not be considered a fit and proper person with whom to deposit such a professional secret?

In the course of the Headmasters' Conference held last week at Bradfield College the question of the Medical Preliminary Examinations was discussed. Dr. Wilson (Lancing) said that there were at present fifty-four different examinations in the United Kingdom which were recognised as taking the place of a preliminary literary examination for people who wanted to be doctors, and eighty-six in various educational bodies throughout the Empire. With the object of introducing order into chaos in regard to the preliminary medical examinations, he moved, "That it is desirablet hat there should be greater uniformity and continuity in the standard of work required for the preliminary medical examinations." To this Mr. Bell moved as an amendment, "That the committee be instructed to communicate with such examining bodies as control admission to professional careers with a view to the establishment of a joint board for the conduct of such examination." The amendment was carried.

At the last ordinary meeting of the Council of the Royal College of Surgeons, a report was received from the committee on the report to the annual meeting of Fellows and members held on November 15th. With reference to Resolution I., it was resolved that the Council are of opinion that it would be most undesirable to re-open the question of representation of members on the Council. With regard to Resolution No. II., it was resolved that, in view of the disability which affects the medical profession, this meeting requests the Council to take any steps in its power towards obtaining a Parliamentary inquiry into the working of the Medical Acts, with a view to their amendment. In answer to Resolution III., it was resolved that the annual report of the Council be sent only to those Fellows and members who apply for it; but that, to save unnecessary trouble to those who desire to receive the report regularly, any Fellow or member may on request have his name entered upon a standing list. With reference to Resolution IV., it was resolved tbat the mover and seconder of this resolution be informed "that the resolution has been laid before the Council."

